# Hypertension in older adults: Assessment, management, and challenges

**DOI:** 10.1002/clc.23303

**Published:** 2019-12-11

**Authors:** Estefania Oliveros, Hena Patel, Stella Kyung, Setri Fugar, Alan Goldberg, Nidhi Madan, Kim A. Williams

**Affiliations:** ^1^ Department of Internal Medicine, Division of Cardiology Rush University Medical Center

**Keywords:** hypertension, older adult, geriatrics, antihypertensive agents, blood pressure monitoring

## Abstract

Hypertension in older adults is related to adverse cardiovascular outcomes, such as heart failure, stroke, myocardial infarction, and death. The global burden of hypertension is increasing due to an aging population and increasing prevalence of obesity, and is estimated to affect one third of the world's population by 2025. Adverse outcomes in older adults are compounded by mechanical hemodynamic changes, arterial stiffness, neurohormonal and autonomic dysregulation, and declining renal function. This review highlights the current evidence and summarizes recent guidelines on hypertension, pertaining to older adults. Management strategies for hypertension in older adults must consider the degree of frailty, increasingly complex medical comorbidities, and psycho‐social factors, and must therefore be individualized. Non‐pharmacological lifestyle interventions should be encouraged to mitigate the risk of developing hypertension, and as an adjunctive therapy to reduce the need for medications. Pharmacological therapy with diuretics, renin‐angiotensin system blockers, and calcium channel blockers have all shown benefit on cardiovascular outcomes in older patients. Given the economic and public health burden of hypertension in the United States and globally, it is critical to address lifestyle modifications in younger generations to prevent hypertension with age.

## 1 INTRODUCTION

The 2018 ACC/AHA^1^ and ESC/ESH guidelines^2^ have different definitions for blood pressure goals: <130/80 mmHg for ACC/AHA and <140/90 mmHg for ESC/ESH. Additional differences arise when addressing the risk and goals in order people at 130/70 to 139/79 mmHg for ESC/ESH but <130/80 mmHg for ACC/AHA. The newer lower thresholds have now included more patients as hypertensives.

HTN is known to have significant effects on cardiovascular (CVD) outcomes such as heart failure, myocardial infarction, and stroke.^3^ The asymptomatic quality of systemic hypertension can delay diagnosis and prompt initiation of optimal therapies. As with many conditions, HTN increases with age, with its prevalence increasing from 27% in patients aged younger than 60 years to 74% in those aged older than 80 years.^4^ The Framingham Heart Study^5^ showed than more than 90% of the participants with a normal blood pressure (BP) at age 55 years eventually develop HTN. Approximately 60% of the population has HTN by 60 years of age and about 65% of men and 75% of women develop high BP by 70 years.^5^ By the year 2060, the projected number of people living age 65 years or older will comprise 25% of the United States (US), of which nearly 20 million will surpass the eighth decade of life. Up to 50% of the people born in the US today will reach their 100th year.^6^ With this rapidly aging population, the prevalence of HTN can only be expected to rise.

Interestingly, several studies that focused on guiding screening and management of HTN traditionally excluded older adults, particularly those over the age of 80 years. Treating elevated BP in older adults has, therefore, remained controversial. However, recent data demonstrates CVD benefits in treating HTN in older adults. This emanates from several key studies, including the UK Prospective Diabetes Study (UKPDS),^7^ the Systolic HTN in the Elderly Program (SHEP),^8^ SPRINT,^9^ the Systolic HTN in Europe trial (Syst‐Eur),^10^ Medical Research Council Working Party,^11^ and the HTN in the Very Elderly Trial (HYVET).^12^ Thus, recognition and appropriate treatment of HTN in older adults should be a priority for physicians. This review article aims to address the challenges in assessment and management of HTN in older adults, as well to address the differences among guidelines and societies.

## 2 PATHOPHYSIOLOGY AND COMPLICATIONS OF HYPERTENSION IN OLDER ADULTS

There are specific underlying mechanisms of HTN in older persons, including mechanical hemodynamic changes, arterial stiffness, neurohormonal and autonomic dysregulation, and the aging kidney.^13^ Aging results in several structural and functional changes in the arterial vasculature. Over time, the arteries stiffen, with fracturing of the elastic lamellae and intimal hyperplasia is seen in the aorta. The stiffened arteries have decreased capacitance, and limited recoil, with subsequent difficulty to accommodate volume changes throughout the cardiac cycle. Both systolic BP (SBP) and diastolic BP (DBP) increase with age, however, after the age of 60 years, the central arterial stiffness predominates, and as a consequence, SBP continues to rise while the DBP declines thereafter.^14^ This results in isolated systolic HTN and a widened pulse pressure. The latter increases with age independently of mean BP or any other determinant factors. Sasaki et al^15^ have shown that NT‐proBNP concentrations may be a marker of not only ventricular dysfunction, but also arterial stiffness in the older population without CVD disease. NT‐proBNP was positively associated with SBP, whereas a U‐shaped association was found between DBP and NT‐proBNP.

Furthermore, there are hemodynamic mechanical changes that alter wave reflection causing a reduction in the aortic elasticity, as well as loss of recoil during diastole. There is also increase in pulse pressure and pulse‐wave velocity. The change in arterial structure causes an increase in the reflected pressure waves added to the forward pressure waves in the ascending aorta that further augments the central SBP.

Over time, endothelial dysfunction occurs, inducing an elevation in endothelin‐1 and decreasing bioavailability of nitric oxide, which affects arterial dilation.^16^ Other neurohormonal mechanisms include a decline in the renin‐angiotensin aldosterone system, with plasma renin levels by age 60 declining to 40% to 60% of younger individuals.^17^ Plasma aldosterone levels also decrease, predisposing individuals to drug‐related hyperkalemia.^18^ Some authors have described increased peripheral plasma norepinephrine related to age,^19^, ^20^ which is thought to be a compensatory mechanism for reduction in beta‐adrenergic responsiveness with aging.^21^

Reduced baroreflex sensitivity with age and loss of artery compliance causes orthostatic hypotension, defined as a reduction in SBP by at least 20 mmHg or DBP by at least 10 mmHg within 3 minutes of standing.^14^ Orthostatic hypotension carries a prevalence of 18% in older adults and is associated with increased falls and cerebrovascular effects.^22^, ^23^ Valbusa et al^22^ showed that beta‐blockers were associated with increased likelihood of developing orthostatic hypotension. The older adults rely on an increased cardiac output due to increased heart rate, as opposed to changes in their stiff arteries to achieve postural homeostasis. There is also evidence of orthostatic HTN and its association with cerebrovascular events in older adults.^24^ A randomized clinical trial suggested that non‐institutionalized elderly targeting a SBP <120 mmHg was not associated with significant increases in orthostatic hypotension.^25^

Postprandial hypotension in geriatric patients is an under‐recognized cause of syncope.^26^ The mechanism is unclear, but it appears to be related to reduced sympathetic response to a meal. Ambulatory BP monitoring and symptoms can give a diagnose. The patient can be advised to increase water intake before eating or substituting six smaller meals daily for three larger meals.^26^ Older adults have an increase frequency of postprandial hypotension. Patients with HF, syncope, Parkinson's disease, end‐stage renal disease on dialysis, autonomic dysfunction can have postprandial hypotension.^27^, ^28^, ^29^, ^30^ Frail older adults with postprandial hypotension increase their postprandial BP and heart rate when walking.^31^

The aging changes in the kidney are increased salt sensitivity due to a decline in the activity of the sodium/potassium and calcium adenosine triphosphate pumps, which prompts vasoconstriction and vascular resistance.^32^ Lastly, HTN in the elderly is also associated with increased risk of ischemic and hemorrhagic strokes,^33^, ^34^ vascular dementia, Alzheimer's disease,^35^ coronary artery disease and events,^36^ atrial fibrillation, chronic kidney disease and retinal diseases.

### 2.1 Diagnosis of hypertension

The diagnosis of hypertension requires measurement of BP in the proper environment under optimum conditions.^1^ It requires that the patient be relaxed in a chair for at least 5 minutes with the arm resting. In order to establish diagnosis, ≥2 readings of elevated BP on ≥2 occasions are needed.^1^ White coat hypertension is more common among elderly patients possibly related to increasing arterial stiffness, thus, ambulatory or out‐of‐office blood pressure readings is important in the subgroup of patients with mildly elevated in‐office BP readings.^2^, ^37^ The 2017 ACC has set a blood pressure reading above ≥130/80 mmhg to be considered hypertensive while the European Society of Hypertension guidelines have maintained a blood pressure reading of ≥140/90 mmhg to be considered to be hypertensive.^38^, ^39^ Since high BP is primarily asymptomatic, structured community programs play an important role in the diagnosis and have proven effective in diagnosis patients unaware they have hypertension.^40^, ^41^

## 3 CURRENT EVIDENCE AND GUIDELINES

Treatment of ISH in older adults was somewhat unclear until randomized studies such as the Systolic Hypertension in the Elderly Program (SHEP),^8^ the Systolic Hypertension in Europe trial (Syst‐Eur),^42^ and the Hypertension in the Very Elderly Trial (HYVET)^43^ showed significant CV benefits on lowering SBP in patients with ISH. All these four trials defined ISH as SBP ≥160 and DBP <90 mmHg. Based on the data mainly from HYVET study, the 2013 European Society of Hypertension/European Society of Cardiology Guidelines^44^ for the management of HTN stated that there is evidence for benefits of lowering the SBP to <150 mmHg in older adults with initial SBP of >160 mmHg. Recently, SPRINT trial added to this evidence base and showed the benefits of a lower BP goal of SBP <120 mmHg in patients aged over 75 years.^25^ Despite this convincing evidence of the benefit of antihypertensive treatment in older adults with HTN, the optimal BP target has remained unclear in this population. This mostly derives from the higher vulnerability of older adults to develop complications from the antihypertensives such as orthostasis, falls and renal dysfunction due to their burden of comorbidities, advanced age and frailty. This was studied in a post hoc analysis of both HYVET^45^ and SPRINT,^25^ however, these studies did not show a relationship between the benefits of antihypertensive treatment and patient frailty. Both studies concluded that antihypertensive treatment goals in frail older patients should be similar to treatment strategies used in the fittest patients. These results were then incorporated in the 2017 Canadian guidelines that proposed to target an SBP of <120 mm Hg for all individuals aged over 75 years.^46^, ^47^ The 2017 American College of Cardiology/American Heart Association (ACC/AHA) guidelines recommend that a BP <130/80 mm Hg should be targeted after the age of 65 years.^47^ However, more recently, the 2018 ESC/ESH guidelines proposed a BP goal of <140/90 mm Hg for individuals older than 65 years.^2^ The 2017 American College of Physicians/American Association of Family Physicians guidelines propose to target a BP <150/90 mm Hg.^48^ Based on these different recommendations regarding the target BP in older adults, it seems clear that there is still debate on optimal BP targets. For most patients, recent evidence suggests that “the lower the better” concept should be considered. However, the Eighth Joint National Committee (JNC 8) defined a higher BP target of 150/90 for people above the age of 60 years. This recommendation was not accepted by all committee members who then published a “minority report” outlining their views of the evidence. It is important to note that there is increased vascular stiffness in older adults that results in higher systolic pressure, lower diastolic pressure and higher pulse pressure. This would probably require different BP lowering strategies compared with younger patients. We recommend the physicians to critically assess the evidence as there are strengths and weaknesses of discussions on BP targets in older adults. Overall, choosing a target blood pressure in older adults with hypertension requires discussion between physician and the patient taking into consideration several factors such as the burden of comorbidity, life expectancy, clinical judgment, and patient preference.

While the SPRINT and HYVET studies support blood pressure control even in frail individuals, analysis of NHANES data by Odden et al suggest that impaired walking speed (a surrogate for frailty) may assist in risk stratifying frail seniors. In slower walkers (<0.8 m/s), elevated SBP and DBP (≥140/90) were not associated with mortality.^49^ Observational data suggests that BP reductions in the frail individuals may be harmful.

A meta‐analysis^50^ identified that between 40 to 69 years of age, each difference of 20 mmHg in the SBP or 10 mmHg increase of DBP is associated with more than 2‐fold difference in stroke‐related death, death due ischemic heart disease and from other vascular etiologies. Differences were about half as extreme between ages 80 to 89 years. Data from The Second National Health and Nutrition Examination Survey (NHANES II) and SHEP trial showed that in the elderly, there is a linear relationship between CVD risk, specifically in stroke, and increasing SBP (the absolute stroke risk in the place group of the SHEP trial was 8.2% over 5 years, compared to the 5% present in the patients that received treatment).^8^, ^51^ The HYVET randomized control trial enrolled individuals age ≥ 80 with a SBP of at 160 mmHg to receive indapamide or placebo, and demonstrated a 21% relative reduction in all‐cause mortality and a 23% relative reduction in CVD mortality after a median of 1.8 years under treatment.^12^, ^43^

Until SPRINT,^9^ most guidelines favored a goal of <140/90 mmHg in patients with chronic kidney disease (CKD). Interestingly, observational studies of CKD cohorts have shown increased mortality at lower SBP and a flat relationship of SBP to event risk in older patients with CKD, which supports the concern of intensive BP management.^52^, ^53^ In the subgroup analysis of the elderly in the SPRINT cohort, frail elderly subjects had benefits from lower BP goals, even in cases with CKD. Both HYVET and SPRINT were stopped early for benefit. It should also be noted that SPRINT excluded those participants that had <110 mmHg standing BP. The investigators that conducted SPRINT Senior^25^ selected 2636 subjects with CVD risk, ≥75 years without stroke, and diabetes mellitus (DM), and randomized them to SBP <140 mmHg vs <120 mmHg.

In the INVEST sub‐study, the adjusted hazard ratio for primary outcomes showed a J‐shaped relationship between each age group with on‐treatment SBP and DBP. The SBP at the hazard ratio nadir increased with aging, highest for the elderly (140 mmHg). Nevertheless, DBP at the hazard ratio nadir was slightly decreased for the very old (70 mmHg).^54^

This evidence has been synthesized into recent guidelines and expert consensus recommendations. The Seventh Joint National Committee (JNC 7)^55^ focused on expert consensus recommendations, whereas later on the Eighth Joint National Committee (JNC 8)^56^ was convened with the intent on generating recommendations based on the best available evidence, prospective randomized trials. The document was commissioned by the NHLBI in 2008, but was not endorsed by AHA, ACC or any of the 41 organizations that endorsed JNC 7, primarily due to the controversial recommendation that the threshold for initiation of therapy and the target of that therapy be set at 150/90 mmHg to avoid overtreatment of the older population. This was criticized by 5 of the writing group's members.^57^

**Figure 1:**
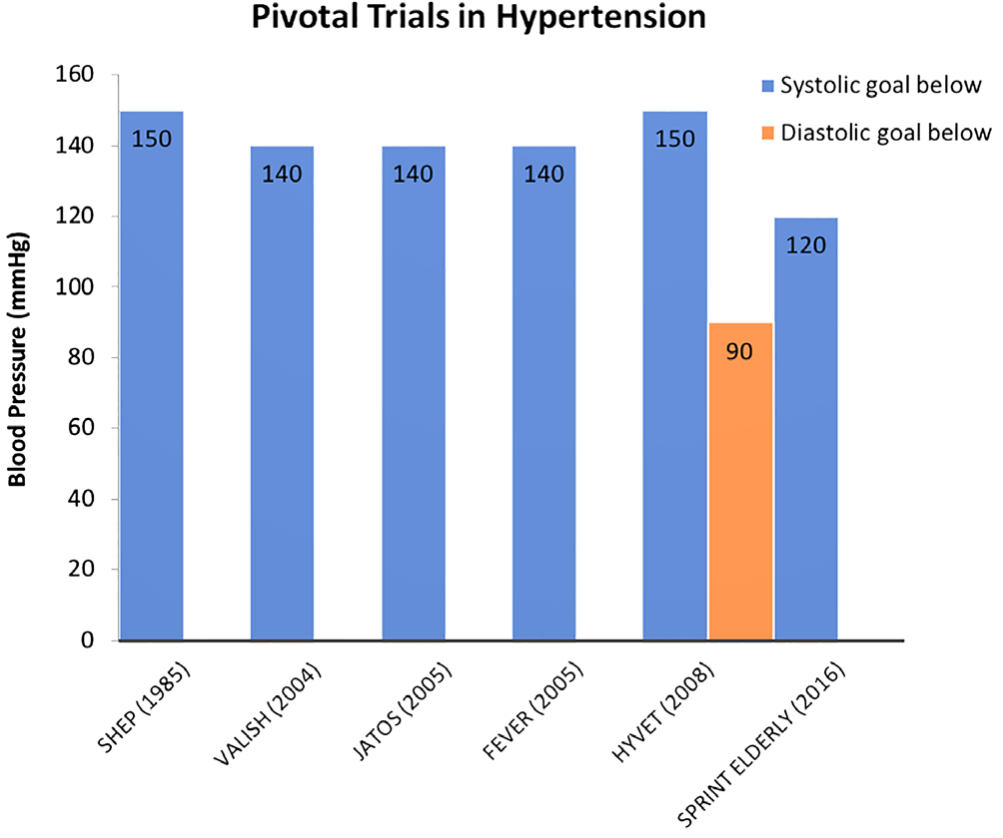
Blood pressure goals in pivotal clinical trials: demonstrated in chronologic order from 1985 to 2016 the systolic and diastolic blood pressure goals in mmHg. We are able to appreciate a remarkable difference in SPRINT ELDERLY compared to prior

The European Society of Cardiology (ESC)/European Society of Hypertension,^2^ the American College of Cardiology/American Heart Association^1^ and the American Society of Hypertension/International Society of Hypertension^58^ have also issued their own documents with differences in targets and management. (Table 1 and Figure 1). Current recommendation from the American College of Physicians and the American Association of Family Practitioners recommend a systolic blood pressure of less than 150 mmHg despite the findings from SPRINT, which represents a 20‐mmHg difference from other societies. Discrepancies like this truly represent a challenge for clinicians.

**Table 1:** Chronological order of the guidelines for the management of high blood pressure in adults and the elderly

Organization	Year	Population	Target Blood Pressure	Considerations for the elderly
Seventh Report of the Joint National Committee on Prevention, Detection, Evaluation, and Treatment of High Blood Pressure (JNC 7)^55^	2003	All adults except those with diabetes or chronic kidney disease Adults with diabetes or chronic kidney disease	<140/90 mmHg <130/80 mmHg	Encourage low sodium and alcohol‐free lifestyle Avoid DBP <50‐60 mmHg
ACCF/AHA 2011 for the elderly^32^	2011			
European Society of Hypertension/European Society of Cardiology (ESH/ECS)^44^	2013	All adults except those with diabetes Adults with diabetes	140‐150 mmHg systolic; consider <140 mmHg if the patient is fit and healthy <85 mmHg DBP	Ages ≥80 years, the patient's mental capacity and physical heath should also be considered if targeting to <140 mmHg Screen for Orthostatic Hypotension before initiating therapy Avoid DBP < 55 mmHg
Prevention, Detection, Evaluation, and Treatment of High Blood Pressure^56^	2014	Adults age < 60 years and those >18 with diabetes or chronic kidney disease	<140/90 mmHg	Adults age ≥ 60 years Goal: <150/90 mmHg If CKD or DM <140/90 mmHg
American Heart Association/American College of Cardiology (ACC)/Centers for Disease Control and Prevention (AHA/ACC/CDC)^59^	2014	All adults	<140/90 mmHg	No age‐specific guidelines
American Society of Hypertension/International Society of Hypertension (ASH/ISH)^58^	2014	Adults ages 18‐79 years	<140/90 mmHg; <130/80 mmHg BP target may be considered in younger adults	Adults ages ≥80 years Diagnose of HTN only if SBP >150 mmHg Goal: <150/90 mmHg
Department of Veterans Affairs/Department of Defense (VA/DoD)^60^	2014	All adults Adults with diabetes	<150/90 mmHg <150/85 mmHg	No age‐specific guidelines
American Heart Association/American College of Cardiology/American Society of Hypertension (AHA/ACC/ASH)^61^	2015	Adults with CAD, except as noted below Adults with MI, stroke, TIA, carotid artery disease, peripheral artery disease or abdominal aortic aneurysm	<140/90 mmHg <130/80 mmHg	Adults ages >80 years Goal: <150/90 mmHg
American College of Cardiology/American Heart Association (ACC/AHA)^1^	2017	All adults	<130/80 mmHg	≥65 + ambulatory: Goal <130 mmHg ≥65 + high burden of comorbidity, limited life expectancy, clinical judgment, patient preference: assess risk/benefit
Hypertension Canada^62^	2017	All adults	<140/90 mmHg	Age 60‐79: goal <140/90 mmHg Age > 79: goal <150/90
American College of Physicians‐elderly^48^	2017			≥60 years: goal <150 mmHg If TIA <140 mmHg
European Society of Hypertension/European Society of Cardiology^2^ (ESH/ECS)	2018	All Adults	<140/90 mmHg	≥60 years: goal <150/90 mmHg, if tolerate <140 mmHg do not change therapy

Abbreviations: CAD, coronary artery disease; CKD, chronic kidney disease; DBP, diastolic blood pressure; DM, diabetes mellitus; MI, myocardial infarction; TIA, transient ischemic attack.

The ACC/AHA guidelines emphasize individualized CV risk assessment with the use of the ASCVD risk calculator, which uses data from a pooled cohort to create personalized risk for each patient. Therefore, an individual with an ASCVD risk calculator score of >10% 10‐year risk will receive more aggressive treatment. While the ESC/ESH guidelines uses instead the SCORE (Systemic Coronary Risk Evaluation) system coupled with risk modifiers and assessment of HTN mediated organ damage, and a patient with >10% 10‐year CV risk will require more aggressive therapy. The SCORE system estimates the risk based on age, sex, tobacco use, total cholesterol and systolic BP and uses European cohort datasets. One of the benefits of using the ESC/ESH guidelines in patients older than 65 years is that the SCORE system was adapted for this group.^63^ Data from Heart Outcomes Prevention Evaluation (HOPE3) showed no benefit of BP lower less than 140/90 mmHg in patients with intermediate risk,^64^ which emphasizes the importance of addressing the CV risks when tailoring BP goals. One size may not fit all, the ESC/ESH guidelines goals may be more feasible in patients with poor vascular compliance, orthostatic hypotension, and high pulse pressures.^33^, ^43^, ^65^

The consensus documents looked at the same evidence and came up with different recommendations. When reviewing the data from the “minority view” paper in Annals there is a statistical type II misinterpretation of the data.^57^ They accepted the null hypothesis (no significant difference in outcome) from prospective trials less than 3 years. The studies lasting more than 3 years (SHEP and FEVER) had more events accumulated for the primary outcome and were all considered positive trials.

## 4 CHALLENGES IN GERIATRIC CARDIOLOGY

The challenge in geriatric cardiology is making decisions not on age alone, but on the overall medical, physical, social, and mental characteristics of the patient. There is definitely a place for personalized medicine. The physician will need to alter current practice to altered pharmacokinetics and pharmacodynamics in aging and be mindful of issues such as cognitive impairment,^66^ competing medical health problems, polypharmacy,^67^ orthostatic hypotension, falls, gait speed,^68^ medication cost and side effects, incontinence, fatigue, visual and auditory limitations, social support, caretaker availability and frailty.^68^, ^69^, ^70^ It is a misconception to assume that advanced age precludes aggressive interventions. It is important to rule out pseudo‐HTN, masked HTN, situational HTN and exclude secondary causes in cases of resistant HTN.

## 5 HYPERTENSION MANAGEMENT IN THE ELDERLY

### 5.1 Non‐pharmacologic interventions

Non‐pharmacologic lifestyle interventions should be encouraged as preventive care for the development of HTN and as adjunctive therapy for established HTN. Current recommendations advocate for regular physical activity, weight control, smoking cessation, stress reduction, and avoidance of excessive alcohol intake.^71^ A heart‐healthy diet, such as the Dietary Approaches to Stop Hypertension (DASH) diet, low carbohydrate, vegetarian, plant‐based and Mediterranean diet. As well as low sodium intake, potassium supplementation (1500 to >3000 mg), calcium or magnesium supplements, consumption of probiotics, fiber, flaxseed, increased protein intake, consumption of garlic, dark chocolate, tea, coffee, and fish oil.^71^ Behavioral therapies including transcendental meditation, yoga, Taiichi and biofeedback have known effect in decreasing BP. Contributing co‐morbidities such as sleep apnea, renal artery stenosis, prostatism, primary aldosteronism should also be addressed. Review the patients medications to ensure they are not on any medications such as nonsteroidal anti‐inflammatory drugs, steroids, angiogenesis inhibitors, tyrosine kinase inhibitors, atypical antipsychotics, antidepressants, amphetamines, hormone replacement therapy, immunosuppressants, and decongestants which can cause HTN.^72^ Use of recreational drugs, caffeine, tea and herbal supplements should also be inquired.

Reduction is sodium intake (approximately 1000 mg per day) and weight loss constitute effective and safe ways to improve BP, as seen in the Trial of Nonpharmacologic Interventions in the Elderly (TONE).^73^ Some observational studies have argued that low salt intake maybe associated with activation of renin‐angiotensin‐aldosterone system with increased sympathetic system activity leads to adverse cardiovascular effects.^74^, ^75^ This was however refuted by a meta‐analysis by Aburto et al, which showed that lowering salt intake to less than 1200 mg per day was safe and beneficial.^76^ It has also been described that intentional weight loss is associated with increased mortality, but in a post hoc analysis of the TONE data, there was no association with an increase in all‐cause mortality in elderly patients that had weight loss and improvement of BP.^73^ The DASH eating plan is the best diet with most data supporting lowering BP.^71^

The recommended physical activity recommended are aerobic exercise (90‐150 minutes per week with achievement of 65% to 75% of heart rate reserve), dynamic resistance (90‐150 minutes per week), or isometric resistance (3 sessions per week for 8 to 10 weeks of 4 × 2 minutes of hand grip, 1‐minute rest, 30% to 40% of maximum voluntary contraction).^71^ Older adults at any submaximal exercise load will exert at a higher maximal capacity and effort than younger individuals,^77^ they may benefit of experienced fitness trainers to define optimal frequency, intensity, and duration of each type of exercise. Aging causes decline in muscle strength and power,^78^ so there are specific goals from the American College of Sports Medicine which recommend in older adults a minimum of 150 minutes of moderate intensity aerobic activity or 75 minutes of vigorous intensity aerobic activity, and two or more non‐consecutive days of moderate‐intensity strengthening activities, with 8 to 10 exercises involving the major group muscles and 8 to 12 repetitions of each exercise.^79^

Dolan et al^80^ highlighted the importance of home ambulatory BP monitoring over clinic BP measurement to predict mortality. In clinical practice home ambulatory monitoring it is a better tool diagnosis and for titration of medications.^81^ The use of telemedicine to manage our patients is a modern tool that will improve our management in older persons that require slow and careful adjustments in their medication, without asking them to overcome the hurdles of transportation, walking and time of having a clinic visit.^82^

### 5.2 Pharmacologic interventions

When medications are needed to manage older adults with uncontrolled HTN, factors to consider prior to selecting a medication include comorbidities, frailty of the patient, ability to follow instructions, complexity of the current regimen, supporting care (ie, spouses and family) and lastly electrolytes and renal function.^1^, ^83^ Thiazide diuretics, angiotensin‐converting‐enzyme inhibitor (ACEI), angiotensin II receptor blockers (ARB), and calcium channel blocker (CCB), have all shown benefit on CVD outcomes in older age patients. Unless clinically indicated by comorbidities, beta blockers should not be used as first line medications because they may worsen CVD outcomes in those over 60 years of age.^11^ Loop diuretics and alpha‐blockers should also be avoided given their association with falls.^84^

In patients with isolated HTN, the ESH/ESC guidelines recommends a calcium antagonist or diuretic in the elderly patients.^83^ This is supported by evidence from the ALLHAT^85^ trial which suggested that low‐dose daily chlorthalidone was most effective in the population and also by the Anglo‐Scandinavian Cardiac Outcomes Trial‐blood‐pressure‐lowering arm (ASCOT‐BPLA) which showed significant overall mortality benefit in subjects aged >60 years when given a combination of CCB and ACE‐I.^86^ Commonly, BP remains uncontrolled on monotherapy and a combination of different agents is needed to achieve adequate BP control. Any of the four first line BP medications can be combined, however based on multiple RCTs RAAS blockers and CCB/thiazide is the preferred combination.^87^ Single pill combination can be utilized with the added benefit of improving medication compliance.^83^ The medications should then be up titrated, with additional medications added as needed to achieve BP targets.

Initiation of any medication should be done with assessment of orthostatic hypotension and gradual titration according to tolerance. Renal function should be assessed to detect possible increases in serum creatinine and reductions in GFR as a result of BP‐related reductions in renal perfusion. Hypokalemia is also an important side effects of diuretics which needs to be monitored. The medical team needs to be cognizant of treatment related side effects which may occur more frequently than reported in clinical trials.

## 6 FUTURE DIRECTIONS

The key component of managing the elderly is to address lifestyle modifications in the younger populations to prevent HTN with aging. Although there is a component just related to aging of the tissues, there is also a critical behavioral component that we can adjust by reducing obesity, incidence of diabetes mellitus, tobacco use, and alcohol consumption.

When a physician sees younger patients, it is important to emphasize the importance of prevention of HTN with lifestyle modification in order to reduce their risk of cognitive impairment, stroke, myocardial infarction, heart failure, renal failure, and other complications. Newer clinical trials addressing polypill could be a solution to increase adherence as it would minimize number tablets. The use of telemedicine, text messages to trend BP medicines and the HoME study will provide answers regarding the use of technology to manage patients from home.^88^ Other investigators are looking into gut microbiota and its effect of hypertension and weight control. New clinical trial evaluating drugs (ie, MK‐8266, fimasartan), for management of HTN in the older adults will soon finalize their recruitment.^88^ Invasive strategies such as renal denervation are also under study.^88^

## 7 CONCLUSION

As Americans are living longer, there will be an increasing burden of HTN in the growing population of elderly patients. While lifestyle changes have the potential to mitigate this, adoption of healthy practices have been lagging in the US population. Education and awareness programs in community centers, place of worship (temples, churches, synagogues and mosques), and businesses, such as the barber shop initiatives will continue to have impact. Screening for blood pressure elevation, improving access to care, and adoption of guideline driven management are keys to reducing the impact of HTN in our elderly patients. Further research is needed in this population, since most clinical trials include a small proportion of persons over age 85 years old. Such trial can help solidify the current recommendations to use >130/80 after lifestyle intervention as the threshold for initiation of therapy and <130/80 at the target of therapy.

## CONFLICT OF INTEREST

The authors declare no potential conflict of interests.

## References

[1] Whelton PK , Carey RM , Aronow WS , et al. 2017 ACC/AHA/AAPA/ABC/ACPM/AGS/APhA/ASH/ASPC/NMA/PCNA Guideline for the Prevention, Detection, Evaluation, and Management of High Blood Pressure in Adults. A Report of the American College of Cardiology/American Heart Association Task Force on Clinical Practice Guidelines. J Am Coll Cardiol. 2018;71(19):e127‐e248. 29146535 10.1016/j.jacc.2017.11.006

[2] Williams B , Mancia G , Spiering W , et al. 2018 ESC/ESH guidelines for the management of arterial hypertension. Eur Heart J. 2018;39(33):3021‐3104. 30165516 10.1093/eurheartj/ehy339

[3] Lawes CM , Vander Hoorn S , Rodgers A . International Society of H. global burden of blood‐pressure‐related disease, 2001. Lancet. 2008;371(9623):1513‐1518. 18456100 10.1016/S0140-6736(08)60655-8

[4] Lloyd‐Jones DM , Evans JC , Levy D . Hypertension in adults across the age spectrum: current outcomes and control in the community. JAMA. 2005;294(4):466‐472. 16046653 10.1001/jama.294.4.466

[5] Franklin SS , Larson MG , Khan SA , et al. Does the relation of blood pressure to coronary heart disease risk change with aging?. The Framingham Heart Study. Circulation. 2001;103(9):1245‐1249. 11238268 10.1161/01.cir.103.9.1245

[6] Goldstein DS . Plasma norepinephrine during stress in essential hypertension. Hypertension. 1981;3(5):551‐556. 7298109 10.1161/01.hyp.3.5.551

[7] King P , Peacock I , Donnelly R . The UK prospective diabetes study (UKPDS): clinical and therapeutic implications for type 2 diabetes. Br J Clin Pharmacol. 1999;48(5):643‐648. 10594464 10.1046/j.1365-2125.1999.00092.x PMC2014359

[8] Prevention of stroke by antihypertensive drug treatment in older persons with isolated systolic hypertension. Final results of the Systolic Hypertension in the Elderly Program (SHEP). SHEP Cooperative Research Group. JAMA. 1991;265(24):3255‐3264. 2046107

[9] Group SR , Wright JT Jr , Williamson JD , et al. A randomized trial of intensive versus standard blood‐pressure control. N Engl J Med. 2015;373(22):2103‐2116. 26551272 10.1056/NEJMoa1511939 PMC4689591

[10] Forette F , Seux ML , Staessen JA , et al. Prevention of dementia in randomised double‐blind placebo‐controlled Systolic Hypertension in Europe (Syst‐Eur) trial. Lancet. 1998;352(9137):1347‐1351. 9802273 10.1016/s0140-6736(98)03086-4

[11] Medical Research Council trial of treatment of hypertension in older adults: principal results. MRC working party. BMJ. 1992;304(6824):405‐412. 1445513 10.1136/bmj.304.6824.405 PMC1995577

[12] Bulpitt CJ , Fletcher AE , Amery A , et al. The Hypertension in the Very Elderly Trial (HYVET). J Hum Hypertens. 1994;8(8):631‐632. 7990098

[13] Lionakis N , Mendrinos D , Sanidas E , Favatas G , Georgopoulou M . Hypertension in the elderly. World J Cardiol. 2012;4(5):135‐147. 22655162 10.4330/wjc.v4.i5.135 PMC3364500

[14] Pinto E . Blood pressure and ageing. Postgrad Med J. 2007;83(976):109‐114. 17308214 10.1136/pgmj.2006.048371 PMC2805932

[15] Sasaki N , Yamamoto H , Ozono R , Fujiwara S , Kihara Y . Association of N‐terminal pro B‐type natriuretic peptide with blood pressure and pulse pressure in elderly people—a cross‐sectional population study. Circ J. 2018;82(8):2049‐2054. 29794411 10.1253/circj.CJ-18-0031

[16] McEniery CM , Yasmin HIR , Qasem A , Wilkinson IB , Cockcroft JR , et al. Normal vascular aging: differential effects on wave reflection and aortic pulse wave velocity: the Anglo‐Cardiff Collaborative Trial (ACCT). J Am Coll Cardiol. 2005;46(9):1753‐1760. 16256881 10.1016/j.jacc.2005.07.037

[17] Epstein M . Aging and the kidney. J Am Soc Nephrol. 1996;7(8):1106‐1122. 8866401 10.1681/ASN.V781106

[18] Fleg JL . Alterations in cardiovascular structure and function with advancing age. Am J Cardiol. 1986;57(5):33C‐44C. 3004186 10.1016/0002-9149(86)91025-8

[19] Veith RC , Featherstone JA , Linares OA , Halter JB . Age differences in plasma norepinephrine kinetics in humans. J Gerontol. 1986;41(3):319‐324. 3700981 10.1093/geronj/41.3.319

[20] Goldstein DS . Plasma norepinephrine in essential hypertension. A study of the studies. Hypertension. 1981;3(1):48‐52. 7203605 10.1161/01.hyp.3.1.48

[21] Ferrara N , Komici K , Corbi G , et al. Beta‐adrenergic receptor responsiveness in aging heart and clinical implications. Front Physiol. 2014;4:396. 24409150 10.3389/fphys.2013.00396 PMC3885807

[22] Valbusa F , Labat C , Salvi P , et al. Orthostatic hypotension in very old individuals living in nursing homes: the PARTAGE study. J Hypertens. 2012;30(1):53‐60. 22080223 10.1097/HJH.0b013e32834d3d73

[23] Rutan GH , Hermanson B , Bild DE , Kittner SJ , LaBaw F , Tell GS . Orthostatic hypotension in older adults. The cardiovascular health study. CHS Collaborative Research Group. Hypertension. 1992;19(6 Pt 1):508‐519. 1592445 10.1161/01.hyp.19.6.508

[24] Kario K , Eguchi K , Hoshide S , et al. U‐curve relationship between orthostatic blood pressure change and silent cerebrovascular disease in elderly hypertensives: orthostatic hypertension as a new cardiovascular risk factor. J Am Coll Cardiol. 2002;40(1):133‐141. 12103267 10.1016/s0735-1097(02)01923-x

[25] Williamson JD , Supiano MA , Applegate WB , et al. Intensive vs standard blood pressure control and cardiovascular disease outcomes in adults aged >/=75 years: a randomized clinical trial. JAMA. 2016;315(24):2673‐2682. 27195814 10.1001/jama.2016.7050 PMC4988796

[26] Luciano GL , Brennan MJ , Rothberg MB . Postprandial hypotension. Am J Med. 2010;123(3):281 e1‐6. 10.1016/j.amjmed.2009.06.026 20193838

[27] Jansen RW , Lipsitz LA . Postprandial hypotension: epidemiology, pathophysiology, and clinical management. Ann Intern Med. 1995;122(4):286‐295. 7825766 10.7326/0003-4819-122-4-199502150-00009

[28] Mehagnoul‐Schipper DJ , Boerman RH , Hoefnagels WH , Jansen RW . Effect of levodopa on orthostatic and postprandial hypotension in elderly Parkinsonian patients. J Gerontol A Biol Sci Med Sci. 2001;56(12):M749‐M755. 11723148 10.1093/gerona/56.12.m749

[29] Mehagnoul‐Schipper DJ , Colier WN , Hoefnagels WH , Verheugt FW , Jansen RW . Effects of furosemide versus captopril on postprandial and orthostatic blood pressure and on cerebral oxygenation in patients > or = 70 years of age with heart failure. Am J Cardiol. 2002;90(6):596‐600. 12231083 10.1016/s0002-9149(02)02562-6

[30] van Kraaij DJ , Jansen RW , Bouwels LH , Hoefnagels WH . Furosemide withdrawal improves postprandial hypotension in elderly patients with heart failure and preserved left ventricular systolic function. Arch Intern Med. 1999;159(14):1599‐1605. 10421283 10.1001/archinte.159.14.1599

[31] Jansen RW . Postprandial hypotension: simple treatment but difficulties with the diagnosis. J Gerontol A Biol Sci Med Sci. 2005;60(10):1268‐1270. 16282557 10.1093/gerona/60.10.1268

[32] Zemel MB , Sowers JR . Salt sensitivity and systemic hypertension in the elderly. Am J Cardiol. 1988;61(16):7H‐12H. 10.1016/0002-9149(88)91098-3 3289354

[33] Perry HM Jr , Davis BR , Price TR , et al. Effect of treating isolated systolic hypertension on the risk of developing various types and subtypes of stroke: the systolic hypertension in the elderly program (SHEP). JAMA. 2000;284(4):465‐471. 10904510 10.1001/jama.284.4.465

[34] Bulpitt CJ , Beckett NS , Cooke J , et al. Results of the pilot study for the hypertension in the very elderly trial. J Hypertens. 2003;21(12):2409‐2417. 14654762 10.1097/00004872-200312000-00030

[35] Rosendorff C , Beeri MS , Silverman JM . Cardiovascular risk factors for Alzheimer's disease. Am J Geriatr Cardiol. 2007;16(3):143‐149. 17483665 10.1111/j.1076-7460.2007.06696.x

[36] Vaccarino V , Holford TR , Krumholz HM . Pulse pressure and risk for myocardial infarction and heart failure in the elderly. J Am Coll Cardiol. 2000;36(1):130‐138. 10898424 10.1016/s0735-1097(00)00687-2

[37] Cobos B , Haskard‐Zolnierek K , Howard K . White coat hypertension: improving the patient‐health care practitioner relationship. Psychol Res Behav Manag. 2015;8:133‐141. 25999772 10.2147/PRBM.S61192 PMC4427265

[38] Whelton PK , Carey RM , Aronow WS , et al. 2017 ACC/AHA/AAPA/ABC/ACPM/AGS/APhA/ASH/ASPC/NMA/PCNA guideline for the prevention, detection, evaluation, and Management of High Blood Pressure in adults: executive summary: a report of the American College of Cardiology/American Heart Association Task Force on Clinical Practice Guidelines. J Am Coll Cardiol. 2018;71(19):2199‐2269. 29146535 10.1016/j.jacc.2017.11.006

[39] Williams B , Mancia G , Spiering W , et al. 2018 Practice guidelines for the management of arterial hypertension of the European Society of Hypertension and the European Society of Cardiology: ESH/ESC Task Force for the Management of Arterial Hypertension. J Hypertens. 2018;36(12):2284‐2309. 30379783 10.1097/HJH.0000000000001961

[40] Chow CK , Teo KK , Rangarajan S , et al. Prevalence, awareness, treatment, and control of hypertension in rural and urban communities in high‐, middle‐, and low‐income countries. JAMA. 2013;310(9):959‐968. 24002282 10.1001/jama.2013.184182

[41] Irazola VE , Gutierrez L , Bloomfield G , et al. Hypertension prevalence, awareness, treatment, and control in selected LMIC communities: results from the NHLBI/UHG Network of Centers of Excellence for Chronic Diseases. Glob Heart. 2016;11(1):47‐59. 27102022 10.1016/j.gheart.2015.12.008 PMC4843831

[42] Staessen JA , Fagard R , Thijs L , et al. Randomised double‐blind comparison of placebo and active treatment for older patients with isolated systolic hypertension. The Systolic Hypertension in Europe (Syst‐Eur) trial investigators. Lancet. 1997;350(9080):757‐764. 9297994 10.1016/s0140-6736(97)05381-6

[43] Beckett NS , Peters R , Fletcher AE , et al. Treatment of hypertension in patients 80 years of age or older. N Engl J Med. 2008;358(18):1887‐1898. 18378519 10.1056/NEJMoa0801369

[44] Mancia G , Fagard R , Narkiewicz K , et al. 2013 ESH/ESC guidelines for the management of arterial hypertension: the Task Force for the Management of Arterial Hypertension of the European Society of Hypertension (ESH) and of the European Society of Cardiology (ESC). Eur Heart J. 2013;34(28):2159‐2219. 23771844 10.1093/eurheartj/eht151

[45] Warwick J , Falaschetti E , Rockwood K , et al. No evidence that frailty modifies the positive impact of antihypertensive treatment in very elderly people: an investigation of the impact of frailty upon treatment effect in the HYpertension in the very elderly trial (HYVET) study, a double‐blind, placebo‐controlled study of antihypertensives in people with hypertension aged 80 and over. BMC Med. 2015;13:78. 25880068 10.1186/s12916-015-0328-1 PMC4404571

[46] Leung AA , Daskalopoulou SS , Dasgupta K , et al. Hypertension Canada's 2017 guidelines for diagnosis, risk assessment, prevention, and treatment of hypertension in adults. Can J Cardiol. 2017;33(5):557‐576. 28449828 10.1016/j.cjca.2017.03.005

[47] Whelton PK , Carey RM , Aronow WS , et al. 2017 ACC/AHA/AAPA/ABC/ACPM/AGS/APhA/ASH/ASPC/NMA/PCNA guideline for the prevention, detection, evaluation, and management of high blood pressure in adults: executive summary: a report of the American College of Cardiology/American Heart Association Task Force on Clinical Practice Guidelines. Hypertension. 2018;71(6):1269‐1324. 29133354 10.1161/HYP.0000000000000066

[48] Qaseem A , Wilt TJ , Rich R , et al. Pharmacologic treatment of hypertension in adults aged 60 years or older to higher versus lower blood pressure targets: a clinical practice guideline from the American College of Physicians and the American Academy of Family Physicians. Ann Intern Med. 2017;166(6):430‐437. 28135725 10.7326/M16-1785

[49] Odden MC , Peralta CA , Haan MN , Covinsky KE . Rethinking the association of high blood pressure with mortality in elderly adults: the impact of frailty. Arch Intern Med. 2012;172(15):1162‐1168. 22801930 10.1001/archinternmed.2012.2555 PMC3537835

[50] Lewington S , Clarke R , Qizilbash N , Peto R , Collins R , Prospective SC . Age‐specific relevance of usual blood pressure to vascular mortality: a meta‐analysis of individual data for one million adults in 61 prospective studies. Lancet. 2002;360(9349):1903‐1913. 12493255 10.1016/s0140-6736(02)11911-8

[51] Brown DW , Giles WH , Greenlund KJ . Blood pressure parameters and risk of fatal stroke, NHANES II mortality study. Am J Hypertens. 2007;20(3):338‐341. 17324749 10.1016/j.amjhyper.2006.08.004

[52] Kovesdy CP , Alrifai A , Gosmanova EO , et al. Age and outcomes associated with BP in patients with incident CKD. Clin J Am Soc Nephrol. 2016;11(5):821‐831. 27103623 10.2215/CJN.08660815 PMC4858482

[53] Weiss JW , Peters D , Yang X , et al. Systolic BP and mortality in older adults with CKD. Clin J Am Soc Nephrol. 2015;10(9):1553‐1559. 26276142 10.2215/CJN.11391114 PMC4559521

[54] Denardo SJ , Gong Y , Nichols WW , et al. Blood pressure and outcomes in very old hypertensive coronary artery disease patients: an INVEST substudy. Am J Med. 2010;123(8):719‐726. 20670726 10.1016/j.amjmed.2010.02.014 PMC3008373

[55] The Seventh Report of the Joint National Committee on Prevention, Detection, Evaluation, and Treatment of High Blood Pressure. Bethesda (MD) 2004. 10.1161/01.HYP.0000107251.49515.c2 14656957

[56] James PA , Oparil S , Carter BL , et al. 2014 evidence‐based guideline for the management of high blood pressure in adults: report from the panel members appointed to the eighth joint National Committee (JNC 8). JAMA. 2014;311(5):507‐520. 24352797 10.1001/jama.2013.284427

[57] Wright JT Jr , Fine LJ , Lackland DT , Ogedegbe G , Dennison Himmelfarb CR . Evidence supporting a systolic blood pressure goal of less than 150 mm hg in patients aged 60 years or older: the minority view. Ann Intern Med. 2014;160(7):499‐503. 24424788 10.7326/M13-2981

[58] Weber MA , Schiffrin EL , White WB , et al. Clinical practice guidelines for the management of hypertension in the community a statement by the American Society of Hypertension and the International Society of Hypertension. J Hypertens. 2014;32(1):3‐15. 24270181 10.1097/HJH.0000000000000065

[59] Go AS , Bauman MA , Coleman King SM , et al. An effective approach to high blood pressure control: a science advisory from the American Heart Association, the American College of Cardiology, and the Centers for Disease Control and Prevention. J Am Coll Cardiol. 2014;63(12):1230‐1238. 24246165 10.1016/j.jacc.2013.11.007

[60] VA/DoD Clinical practice guidelines for the diagnosis and management of hypertension in the primary care setting. 2014.

[61] Rosendorff C , Lackland DT , Allison M , et al. Treatment of hypertension in patients with coronary artery disease: a scientific statement from the American Heart Association, American College of Cardiology, and American Society of Hypertension. Circulation. 2015;131(19):e435‐e470. 25829340 10.1161/CIR.0000000000000207 PMC8365343

[62] Lamb SA , Al Hamarneh YN , Houle SKD , Leung AA , Tsuyuki RT . Hypertension Canada's 2017 guidelines for diagnosis, risk assessment, prevention and treatment of hypertension in adults for pharmacists: an update. Can Pharm J (Ott). 2018;151(1):33‐42. 29317935 10.1177/1715163517743525 PMC5755821

[63] Piepoli MF , Hoes AW , Agewall S , et al. 2016 European Guidelines on cardiovascular disease prevention in clinical practice: the Sixth Joint Task Force of the European Society of Cardiology and Other Societies on Cardiovascular Disease Prevention in Clinical Practice (constituted by representatives of 10 societies and by invited experts) developed with the special contribution of the European Association for Cardiovascular Prevention & Rehabilitation (EACPR). Eur Heart J. 2016;37(29):2315‐2381. 27222591 10.1093/eurheartj/ehw106 PMC4986030

[64] Lonn EM , Bosch J , Lopez‐Jaramillo P , et al. Blood‐pressure lowering in intermediate‐risk persons without cardiovascular disease. N Engl J Med. 2016;374(21):2009‐2020. 27041480 10.1056/NEJMoa1600175

[65] Beddhu S , Chertow GM , Cheung AK , et al. Influence of baseline diastolic blood pressure on effects of intensive compared with standard blood pressure control. Circulation. 2018;137(2):134‐143. 29021322 10.1161/CIRCULATIONAHA.117.030848 PMC5760457

[66] Sabayan B , van Vliet P , de Ruijter W , Gussekloo J , de Craen AJ , Westendorp RG . High blood pressure, physical and cognitive function, and risk of stroke in the oldest old: the Leiden 85‐plus study. Stroke. 2013;44(1):15‐20. 23132780 10.1161/STROKEAHA.112.663062

[67] American Geriatrics Society Beers Criteria Update Expert P . American Geriatrics Society updated beers criteria for potentially inappropriate medication use in older adults. J Am Geriatr Soc. 2012;60(4):616‐631. 22376048 10.1111/j.1532-5415.2012.03923.x PMC3571677

[68] Odden MC , Moran AE , Coxson PG , Peralta CA , Goldman L , Bibbins‐Domingo K . Gait speed as a guide for blood pressure targets in older adults: a modeling study. J Am Geriatr Soc. 2016;64(5):1015‐1023. 27225357 10.1111/jgs.14084 PMC5030071

[69] Abellan van Kan G , Rolland Y , Houles M , Gillette‐Guyonnet S , Soto M , Vellas B . The assessment of frailty in older adults. Clin Geriatr Med. 2010;26(2):275‐286. 20497846 10.1016/j.cger.2010.02.002

[70] Odden MC , Beilby PR , Peralta CA . Blood pressure in older adults: the importance of frailty. Curr Hypertens Rep. 2015;17(7):55. 26068656 10.1007/s11906-015-0564-y PMC5639920

[71] Whelton PK , Carey RM , Aronow WS , et al. ACC/AHA/AAPA/ABC/ACPM/AGS/APhA/ASH/ASPC/NMA/PCNA guideline for the prevention, detection, evaluation, and Management of High Blood Pressure in adults: executive summary: a report of the American College of Cardiology/American Heart Association task force on clinical practice guidelines. Hypertension. 2017;2017:2199‐2269. 10.1161/HYP.0000000000000066 29133354

[72] Grossman A , Messerli FH , Grossman E . Drug induced hypertension—an unappreciated cause of secondary hypertension. Eur J Pharmacol. 2015;763(Pt A):15‐22. 26096556 10.1016/j.ejphar.2015.06.027

[73] Shea MK , Nicklas BJ , Houston DK , et al. The effect of intentional weight loss on all‐cause mortality in older adults: results of a randomized controlled weight‐loss trial. Am J Clin Nutr. 2011;94(3):839‐846. 21775558 10.3945/ajcn.110.006379 PMC3155925

[74] Alderman MH . Reducing dietary sodium: the case for caution. JAMA. 2010;303(5):448‐449. 20124541 10.1001/jama.2010.69

[75] Batuman V . Salt and hypertension: why is there still a debate? Kidney Int Suppl. 2013;3(4):316‐320. 10.1038/kisup.2013.66 PMC4089608 25019011

[76] Aburto NJ , Ziolkovska A , Hooper L , Elliott P , Cappuccio FP , Meerpohl JJ . Effect of lower sodium intake on health: systematic review and meta‐analyses. BMJ. 2013;346:f1326. 23558163 10.1136/bmj.f1326 PMC4816261

[77] Lee PG , Jackson EA , Richardson CR . Exercise prescriptions in older adults. Am Fam Physician. 2017;95(7):425‐432. 28409595

[78] Costello E , Kafchinski M , Vrazel J , Sullivan P . Motivators, barriers, and beliefs regarding physical activity in an older adult population. J Geriatr Phys Ther. 2011;34(3):138‐147. 21937904 10.1519/JPT.0b013e31820e0e71

[79] American College of Sports M , Chodzko‐Zajko WJ , Proctor DN , et al. American College of Sports Medicine position stand. Exercise and physical activity for older adults. Med Sci Sports Exerc. 2009;41(7):1510‐1530. 19516148 10.1249/MSS.0b013e3181a0c95c

[80] Dolan E , Stanton A , Thijs L , et al. Superiority of ambulatory over clinic blood pressure measurement in predicting mortality: the Dublin outcome study. Hypertension. 2005;46(1):156‐161. 15939805 10.1161/01.HYP.0000170138.56903.7a

[81] Agarwal R , Bills JE , Hecht TJ , Light RP . Role of home blood pressure monitoring in overcoming therapeutic inertia and improving hypertension control: a systematic review and meta‐analysis. Hypertension. 2011;57(1):29‐38. 21115879 10.1161/HYPERTENSIONAHA.110.160911

[82] Czaja SJ , Lee CC , Arana N , Nair SN , Sharit J . Use of a telehealth system by older adults with hypertension. J Telemed Telecare. 2014;20(4):184‐191. 24803275 10.1177/1357633X14533889 PMC4982837

[83] Zanchetti A , Dominiczak A , Coca A , et al. 2018 ESC/ESH Guidelines for the management of arterial hypertension. Eur Heart J. 2018;39(33):3021‐3104. 30165516 10.1093/eurheartj/ehy339

[84] Corrao G , Mazzola P , Monzio Compagnoni M , et al. Antihypertensive medications, loop diuretics, and risk of hip fracture in the elderly: a population‐based cohort study of 81,617 Italian patients newly treated between 2005 and 2009. Drugs Aging. 2015;32(11):927‐936. 26589307 10.1007/s40266-015-0306-5

[85] Officers A . Coordinators for the ACRGTA, lipid‐lowering treatment to prevent heart Attack *T. major* outcomes in high‐risk hypertensive patients randomized to angiotensin‐converting enzyme inhibitor or calcium channel blocker vs diuretic: the antihypertensive and lipid‐lowering treatment to prevent heart attack trial (ALLHAT). JAMA. 2002;288(23):2981‐2997. 12479763 10.1001/jama.288.23.2981

[86] Dahlof B , Sever PS , Poulter NR , et al. Prevention of cardiovascular events with an antihypertensive regimen of amlodipine adding perindopril as required versus atenolol adding bendroflumethiazide as required, in the Anglo‐Scandinavian cardiac outcomes trial‐blood pressure lowering arm (ASCOT‐BPLA): a multicentre randomised controlled trial. Lancet. 2005;366(9489):895‐906. 16154016 10.1016/S0140-6736(05)67185-1

[87] Jamerson K , Weber MA , Bakris GL , et al. Benazepril plus amlodipine or hydrochlorothiazide for hypertension in high‐risk patients. N Engl J Med. 2008;359(23):2417‐2428. 19052124 10.1056/NEJMoa0806182

[88] Clinical Trial Finder. Available from: https://www.cardiosmart.org/Heart-Conditions/High-Blood-Pressure/The-Research/Clinical-Trial-Finder.

